# Obituary: Hans Gross

**DOI:** 10.1093/nar/gkaa152

**Published:** 2020-04-01

Hans J. Gross, Prof. Emeritus and former Head of Biochemistry at the University of Würzburg died 6 August 2019 at the age of 83. Hans was involved with NAR from the journal's beginning in 1974, and published in the first issue (*Nucleic Acids Res*., (1974) 1, 35–44). In 1983, Hans joined NAR’s Editorial Board before becoming an Executive Editor in 1992 until 2010, and sensitively handled well over a thousand papers, dealing with authors and referees with his characteristic charm and gentle good humor. Trained in chemistry, Hans switched to biochemistry and made major contributions to RNA structure, enzymology and function.


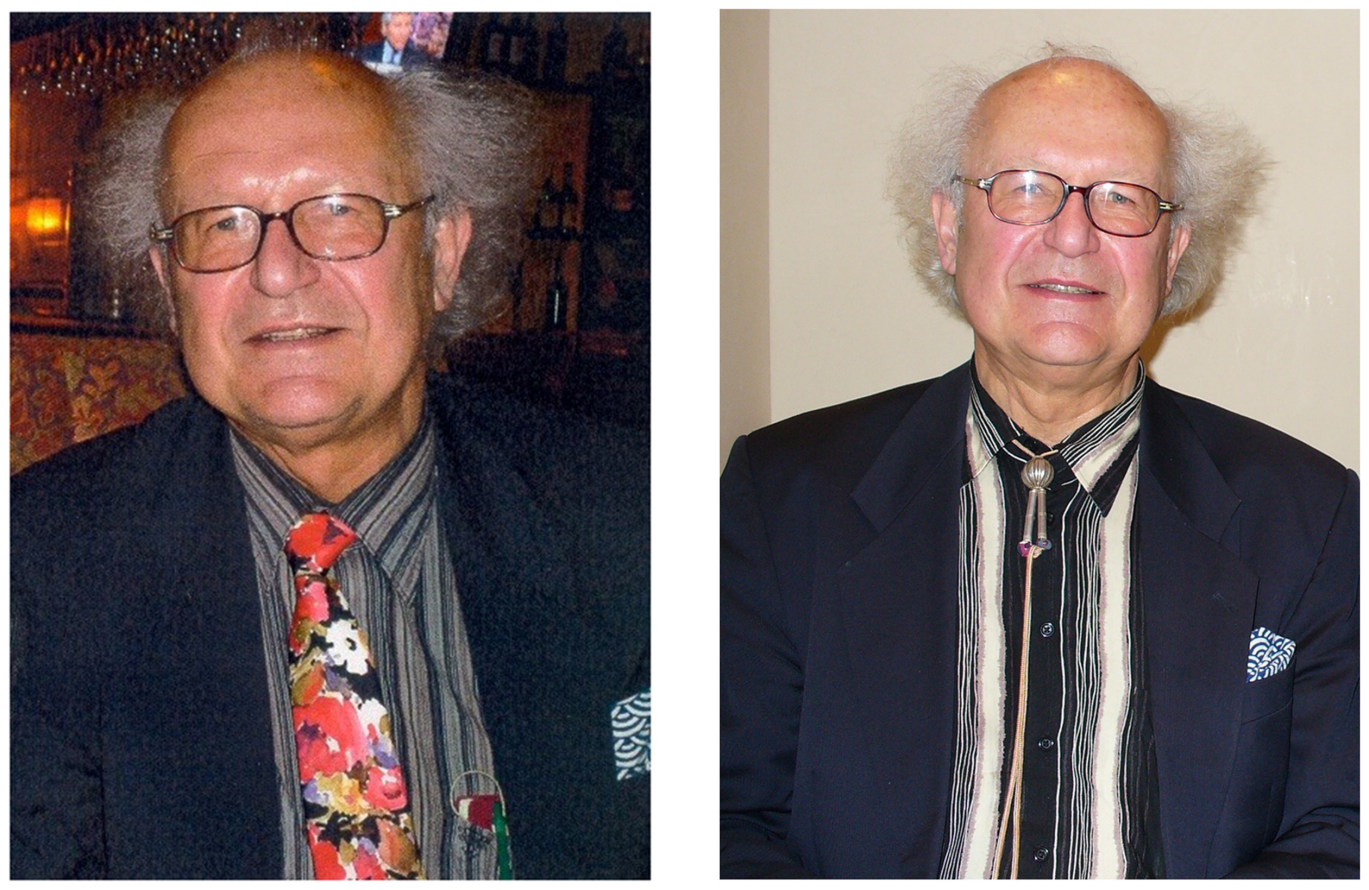


Hans was born on 11 April 1936 in the German province of Silesia, which is now part of Poland. His childhood was a difficult one. When he was 7 years old, his father was killed during the Second World War in Russia. After the war ended, Silesia was ceded back to Poland and Hans’s mother moved to a small town in Bavaria, Germany. School was 12 km away and Hans rode his bicycle each way to school every day of the week. In spite of this arduous daily commute, he did well in school to get into the ‘Ludwig Maximillians University’ in Munich and to obtain a diploma in Chemistry in 1962.

Hans did his PhD research with Heinz Dannenberg at the Max Planck Institute of Biochemistry, also in Munich, on aromatic reactions of steroids. The director of the Institute was Adolf Butenandt, a mentor of Dannenberg, known for his discovery of the steroid hormones estrogen, progesterone and androsterone, for which he was awarded the Nobel Prize in Chemistry. Hans stayed on at the Max Planck Institute for postdoctoral research with Dannenberg for two more years and then went to work with H. Gobind Khorana at the University of Wisconsin in the USA. His stay in Wisconsin was to have a lasting effect; Hans was introduced to tRNAs and he developed a lifelong interest in tRNAs and nucleic acids, an area in which he worked for the rest of his scientific career. In 1968, Hans returned to the Max Planck Institute, now in Martinsried outside of Munich, as an independent investigator and in 1980 moved to the University of Würzburg as Professor and Head of the Institute of Biochemistry, a position he would hold until 2003, when he retired.

Elected to the European Molecular Biology Organization in 1980, Hans was prolific as a scientist and made many important contributions. His first major contribution was the sequence and structural analysis in 1978 of viroid RNA. Viroids are plant pathogens, which are small RNA molecules (246–371-nt long) and are of much interest since they are different from plant viruses, which have much longer RNAs and code for many proteins. Hans’ work published in collaboration with Heinz Sänger established the sequence for the first time of a 359 nt long viroid RNA, at a time when it was extremely difficult to sequence RNA molecules of this size, particularly those RNAs obtainable only in miniscule amounts and not available in radioactively labeled form. Hans’ work also showed that the viroid RNA was circular with a very tight rod like secondary structure and with no potential for coding proteins of reasonable size.

Hans’ next major contribution was on RNA splicing. He discovered in wheat germ extracts an RNA ligase with novel and—at the time—unexpected properties. His work showed that the ligase could circularize RNA molecules carrying specific 5′- and 3′- termini and the ligase turned out to be involved in tRNA splicing in plants, yeasts and other organisms. The same ligase could also convert a linear viroid RNA into a circular one, suggesting its possible involvement also in the circularization step of viroid RNA replication.

Hans also published many important papers on tRNA biochemistry, structure and function including identity elements on the tRNA that inserts selenocysteine into proteins in mammalian cells. tRNAs were in many ways his first love in science. One of his students said that the mantra in Hans’s lab was ‘the most popular four-letter word in science is the word tRNA’. Some of Hans’ work on tRNA was also carried out in collaboration with his longtime collaborator and scientific partner Hildburg Beier, a faculty colleague in Würzburg.

Hans had a broad interest in science. After retiring and closing his lab, he continued to work with another research group in Würzburg, this time on the innate ability of honeybees for number recognition without counting.

Over years in teaching and research, Hans trained many scientists, many of whom are active in academia and in biotech industry. He was a great mentor to students and postdocs in his lab. He supported them wholeheartedly and took great satisfaction in their accomplishments. They, in turn, admired him and were most thankful to him for his support and for his guidance.

Hans often used subtle humor and storytelling effectively to make a point in scientific communication and in teaching. An example comes from his description of work on sequence analysis of viroid RNAs. This required the development of methods for purification of viroid RNAs, which are present in miniscule amounts in infected plants and assays for infectivity of viroid RNAs took weeks. One of us still remembers a slide that Hans showed at a meeting over 45 years ago, consisting of a row of tomato plants, most of them vibrant green, tall and healthy looking, except for those toward the middle inoculated with viroid RNA, which were shriveled, short and sad looking. The slide left no doubt about infectivity of the short viroid RNAs on their own.

One of us also remembers Hans talking about mushrooms at a family dinner event, and Hans saying ‘you can eat any type of mushroom (a pause), but (another pause), some only once’. This led to the question of why and Hans would go on to the topic of toxins, the nature of the toxins and how they worked. As a youngster, Hans loved hiking in the woods and learnt to forage for edible mushrooms.

Outside of research and teaching, Hans also made important contributions toward publication of scientific journals and books. He was an Executive Editor of Nucleic Acids Research for close to two decades and played an important role in the development of the journal. Not only did he pursue the normal responsibilities of making decisions on acceptance or rejection of manuscripts, but he was always helpful in suggesting improvements and notably in telling authors when they could ignore the remarks of a reviewer, who seemed unnecessarily partisan. At the Annual Editorial Board Meetings, Hans would often come up with ideas aimed at improving the journal, which others had not considered previously. One of us remembers many occasions when Hans’s sense of humor and fun greatly enlivened the meetings and encouraged an irreverence that might not have otherwise surfaced and also what a pleasure it was to interact with Hans at these meetings. From 2001 to 2010, Hans was also the editor of the series of topical monographs on Nucleic Acids and Molecular Biology published by Springer Verlag.

As a person, Hans was physically strong, perhaps from his days as a lumberjack in the forests of Finland during holiday periods from the University. He was also an avid gardener (he taught one of us how to graft one branch of a fruit tree to another tree), an excellent cook and he was always full of life. He was a loving father to his daughters. Above all, Hans was a most caring, kind and a compassionate person, always willing to help those in need.

Hans was also an avid fisherman, almost quirky at times. While a postdoc at Wisconsin, during heavy thunderstorms, when most rush to shelters underground, Hans was known to tie an old wooden boat to the top of his small VW beetle car, and race to a deep lake about an hour drive outside of Madison to fish. He was a strong believer in the fish going for the bait during stormy weather.

To both of us, who knew him for so long (one over a period of more than 50 years), and admired him for his accomplishments in science and for who he was as a person, Hans was a dear friend and a wonderful colleague to be around and we miss him deeply. The community at large has also lost a scientist, who made many important contributions in science and in education.

Uttam L. RajBhandary

Richard J. Roberts

Uttam L. RajBhandary is in the Department of Biology at the Massachusetts Institute of Technology, Cambridge, MA 02139, USA and Richard J. Roberts is in New England Biolabs, Inc; Ipswich, MA 01938, USA.

